# A Rare Case of Pulmonary Alveolar Microlithiasis

**DOI:** 10.7759/cureus.23769

**Published:** 2022-04-02

**Authors:** Divya Madhala, Meera Govindarajan, Rajkumar Kulasekaran

**Affiliations:** 1 Pathology, Sri Ramachandra Institute of Higher Education and Research, Chennai, IND; 2 Pathology, R&D Histopathology Lab, Chennai, IND; 3 Pulmonology, Fortis Malar Hospital, Chennai, IND

**Keywords:** biopsy, bronchoscopy, pathology, calcifications, microliths

## Abstract

Pulmonary alveolar microlithiasis is a rare, inherited disease affecting males usually. We present a case of a 32-year-old male who had shortness of breath for two years. The chest x-ray showed fine micronodules in a sandstorm-like pattern distributed bilaterally throughout the lungs, and a high-resolution computed tomography (HRCT) showed diffuse bilateral calcification of the lungs. Following this, a bronchoscopic biopsy was done to confirm the diagnosis.

## Introduction

Pulmonary alveolar microlithiasis (PAM) is a rare, inherited autosomal recessive disorder affecting both sexes and associated with calcospherites involving the lungs. The calcospherites composed of calcium phosphate microliths accumulate in the alveolar spaces of the lungs resulting in widespread damage to the alveoli and the surrounding lung tissues. Follow-up of these patients diagnosed with PAM revealed a very slow progression of the disease and survival for a longer period. Here, we present a 32-year-old male patient diagnosed with PAM at the third stage of the disease where we could avoid a lung transplant and treat it with medication.

## Case presentation

A 32-year-old male patient from Bahrain with complaints of breathlessness for two years was admitted to a tertiary care hospital. The family history of the patient revealed that his parents had a third-degree consanguineous marriage. On clinical examination, his pulse and temperature were found to be normal. A respiratory system examination revealed that his respiratory rate was mildly increased. His SO_2_ was 91% and forced expiratory volume (FEV) was 50%. Lung function tests showed a mild decrease in the capacity of the lungs. Other system examinations were normal.

**Figure 1:**
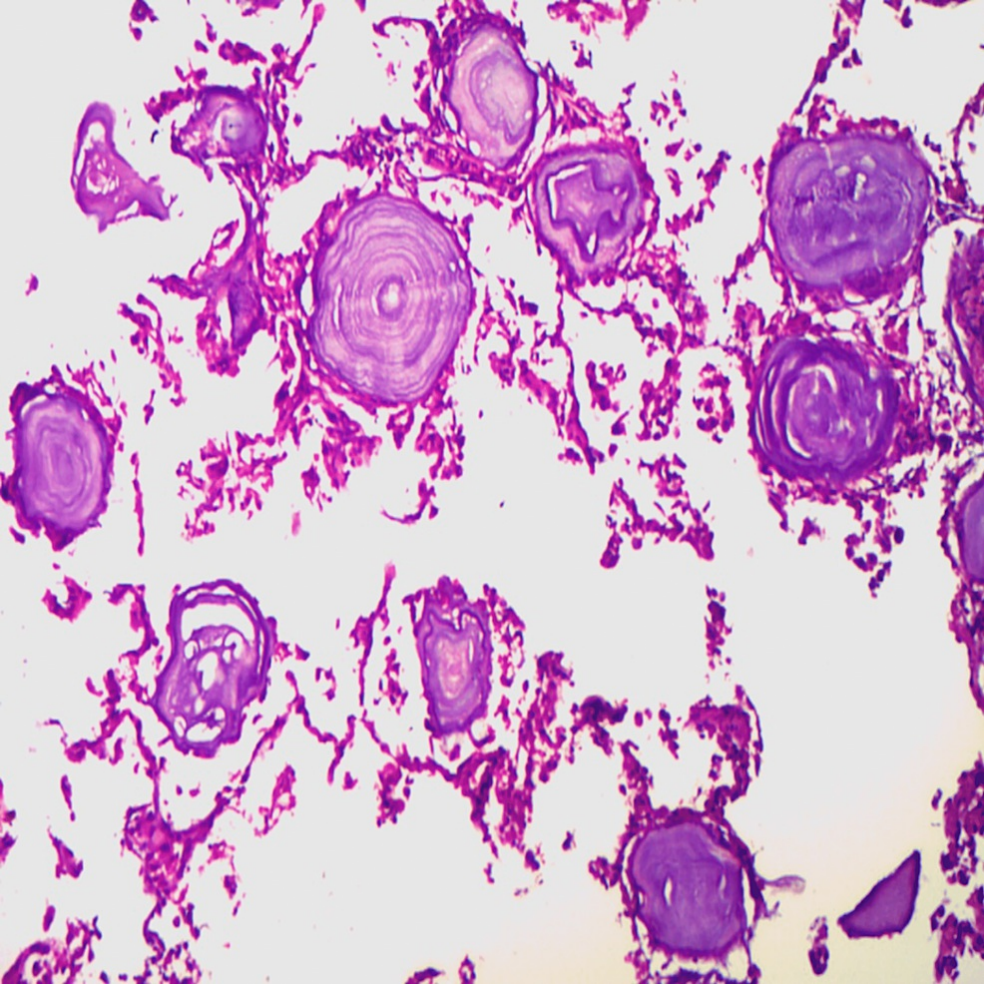
Histopathological examination shows dystrophic calcified bodies

A chest x-ray was advised, which showed fine micronodules in a sandstorm-like pattern distributed bilaterally throughout the lungs. High-resolution computed tomography (HRCT) showed diffuse bilateral calcification of the lungs. Following this, a bronchoscopic biopsy of the lung was done. The biopsy material fixed in 10% formalin was sent for histopathological examination. The sections of the alveoli revealed complete loss of pneumocytes and alveolar lining. The lumen was replaced with dystrophic calcified bodies (Figure 1). Minimal fibrosis was present between the alveoli.

## Discussion

PAM is a slowly progressing autosomal recessive disorder. PAM was first described histopathologically by Harbitz in 1918. Since then, more than 1000 cases have been reported in the world literature [1]. The disease though present worldwide is more prevalent in Asian countries. An increased number of cases were seen in Turkey followed by China, Japan, and India [2]. PAM has a mildly increased male predominance [2]. Although PAM has been reported in all age groups, it typically occurs in the second or third decade of life [2].

PAM is thought to be an inherited familial disorder. Senyiğit et al. described six cases of PAM from the same family [3]. In many cases, the parents of these affected patients were cousins who have had a consanguineous marriage [4]. This is seen typically in our case where the parents of the 32-year-old male were cousins.

The pattern of inheritance is autosomal recessive. There is homozygous loss of function mutations in the gene *SCL34A2 *that encodes the protein type IIb sodium‐phosphate cotransporter. This protein is involved in phosphate homeostasis. The microlith formation is due to the chelation of calcium phosphate, which occurs as a result of reduced reuptake of phosphate by the type llb sodium-phosphate cotransporter in the apical membrane of type ll alveolar cells [5]. The lower lobes of the lungs are affected first due to the deposition of calcium that forms tiny calculi, and this may later progress to involve the whole lungs for 20-40 years. The size of these microliths may range from 0.01 to 2.8 mm. They occupy the entire alveolar space and, in the later stages, induce fibrosis of the alveolar wall with interstitial thickening [4]. These calcium deposits are also seen in extrapulmonary regions including the heart, male genital tract, prostate, and kidneys.

Radiologically, there are four stages. In the first stage, the chest x-ray does not show any changes related to PAM. This stage is seen in children. In the second stage, there are diffusely scattered calcific nodules < 1 mm in size, but some can reach up to 2-4 mm. This gives the typical snowstorm appearance. These findings are present in the second decade of life. In the third stage, there is an increase in the opacifications and some interstitial thickening. In the fourth stage, there is an intense calcification of the interstitium and pleural serosa along with an increase in the size of calcium deposits. The last two stages are seen later in life. The chest x-ray findings of our patient were similar to the third stage of PAM [6].

Histopathological examination shows microliths that are composed of calcium and phosphorus in the ratio of 2:1 with varying amounts of iron, magnesium, copper, and potassium. They have a central granular or amorphous nucleus surrounded by concentric calcareous lamellae. Fibrosis of the alveolar wall is also noted. These microliths that range from 50 to 1000 microns stain positive with periodic acid-Schiff staining [2,6].

At present, there is no medical treatment available to treat PAM, but the overall prognosis of this inherited disease is very good as a result of its slow progression to end-stage disease. Palliative treatments include bronchodilators, systemic corticosteroids, calcium chelating agents, and bronchopulmonary lavage along with lung physiotherapy. The only treatment of choice for the end-stage disease remains to be a lung transplant [2]. Our patient is at present on medication with a yearly follow-up and lung function tests.

## Conclusions

PAM, a rare, inherited autosomal recessive disorder, is seen in cultures where consanguineous marriages are more common. Patients usually develop the late stage of disease in the third and fourth decades of life when transplant is the only modality available for treatment. Further research into molecular and genetic analysis could possibly introduce gene therapy with the correction of the gene *SCL34A2*.
